# Supplementary material for: Splice-Junction-Based Mapping of Alternative Isoforms in the Human Proteome
Source: Cell Rep. Author manuscript; Available in PMC 2020 Jan 15. (PMC6961840; doi:10.1016/j.celrep.2019.11.026)
Supplement: 2 [file NIHMS1546469-supplement-2.zip › DF1/PXD006675/LeftCoronaryArtery/LeftCoronaryArtery_12_BBS1_EGQSAPLLSAHVNMPGSEGLAAPNRPLNPE.pdf]

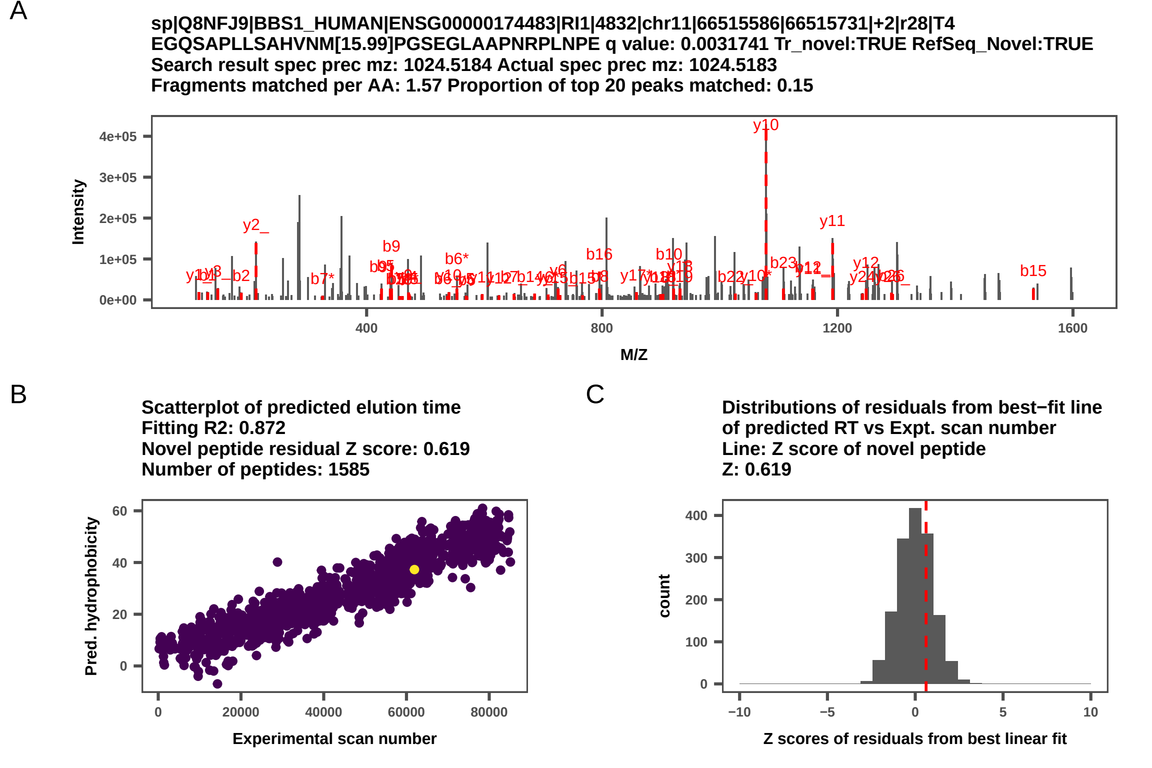
A
sp|Q8NFJ9|BBS1_HUMAN|ENSG00000174483|RI1|4832|chr11|66515586|66515731|+2|r28|T4
EGQSAPLLSAHVNM[15.99]PGSEGLAAPNRPLNPE q value: 0.0031741 Tr_novel:TRUE RefSeq_Novel:TRUE
Search result spec prec mz: 1024.5184 Actual spec prec mz: 1024.5183
Fragments matched per AA: 1.57 Proportion of top 20 peaks matched: 0.15
Intensity
4e+05
3e+05
2e+05
1e+05
0e+00
y10
y2_
b9
y11
b16
b10
b6*
b23
y12
b15
b2
b7*
y6
b8
400
800
1200
1600
M/Z
B
Scatterplot of predicted elution time
Fitting R2: 0.872
Novel peptide residual Z score: 0.619
Number of peptides: 1585
Pred. hydrophobicity
60
40
20
0
0
20000
40000
60000
80000
Experimental scan number
C
Distributions of residuals from best−fit line
of predicted RT vs Expt. scan number
Line: Z score of novel peptide
Z: 0.619
count
400
300
200
100
0
−10
−5
0
5
10
Z scores of residuals from best linear fit
